# Retroperitoneal abscess with pylephlebitis caused by lumbar acupuncture: a case report

**DOI:** 10.1186/s12893-019-0613-6

**Published:** 2019-10-16

**Authors:** Hayemin Lee, Kiyoung Sung, Jinbeom Cho

**Affiliations:** Department of Surgery, Bucheon St. Mary’s Hospital, College of Medicine, The Catholic University of Korea, 327, Sosa-ro, Bucheon-si, Gyeonggi-do 14647 South Korea

**Keywords:** Retroperitoneal abscess, Acupuncture, Sepsis

## Abstract

**Background:**

Retroperitoneal abscess (RA) is an unusual life-threatening disease that has insidious and occult presentations. Although the incidence of this disease is low, diagnosis and treatment are challenging due to its nonspecific presentation and the complex anatomy of the retroperitoneal space. Recently, we experienced one case of a RA with extensive thrombophlebitis of the portal venous system.

**Case presentation:**

An 80-year-old male presented to the emergency room with symptoms and signs of septic shock; however, the decision making for diagnosis and treatment was difficult, as no clinical and radiological evidence supported key findings regarding the origin of sepsis. Although this patient eventually recovered after surgical drainage, we suggested that more straightforward diagnostic and treatment procedures were required in this patient to avoid possible critical complications. Through a retrospective review of operative findings, patient history, and microbiology, we found that the RA in this patient was caused by lumbar acupuncture, which is usually performed for the management of chronic back pain with long needles.

**Conclusion:**

Early surgical intervention should be considered for RA whenever the patient does not respond to broad-spectrum antibiotic treatment. Acupuncture is a possible cause of otherwise unexplained soft tissue infections, such as RA, especially in Asian countries.

## Background

Retroperitoneal abscess (RA) is an uncommon disease that is mainly caused by perinephric inflammation, infections of the gastrointestinal tract, and postoperative complications [1]. Patients usually have comorbidities, such as diabetes mellitus, malignancy, and renal failure. These characteristics seem to contribute to a fatal outcome of this disease. We recently treated a patient who exhibited septic shock of unknown origin. This patient was eventually confirmed to have RA through several diagnostic work-ups and recovered after surgical drainage; however, definitive treatment was delayed due to diagnostic uncertainty, and the outcome could have been fatal. Furthermore, lumbar acupuncture might have caused RA in this patient. Acupuncture, which is used in traditional medicine, is an accepted treatment for chronic musculoskeletal pain [2]. Acupuncture is recommended to be performed by well-trained healthcare professionals.

Here, we report on this rare but critical case to discuss optimal diagnostic and treatment strategies for RA. This is the first reported case of RA caused by acupuncture.

## Case presentation

**Fig. 1:**
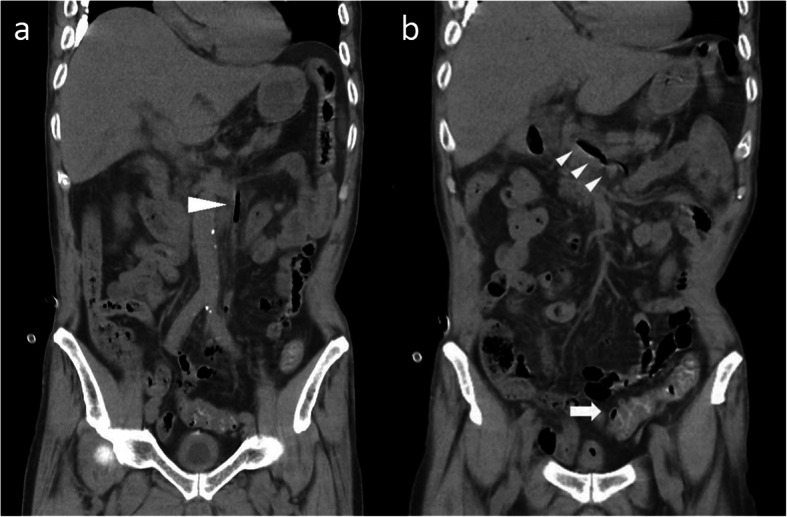
Nonenhanced CT at admission showed extensive thrombophlebitis of the portal venous system (**a** and **b** arrowhead) and sigmoid colon wall thickening with diverticulum (**b** arrow)

An 80-year-old male patient was admitted to the emergency medical center of our hospital based on a complaint of myalgia and abdominal pain. According to the patient and his daughter, the patient had no known comorbidities, including psychiatric disorders, immune deficiency or trauma-related problems. The patient was hemodynamically unstable; he was hypotensive, and his body temperature was increased to 40.7 °C upon the first examination. As the patient exhibited jaundice with abnormal laboratory findings (total bilirubin, 5.46 mg/dL; aspartate transaminase, 251 U/L; alanine transaminase, 143 U/L), bedside biliary ultrasonography was immediately performed, and the results showed no abnormal findings with regard to the biliary system. A subsequent computed tomography (CT) scan of the abdomen showed diffuse wall thickening and several diverticula of the sigmoid colon with multiple air bubbles in the portal venous system (Fig. 1a and b). Nevertheless, we found no abnormalities in the biliary system, such as a gallstone or cholelithiasis; however, no further information could be established via the CT scan because the study was performed without contrast enhancement due to the decreased renal function of the patient. The initial diagnosis made at the time of admission was septic shock caused by atypical biliary disease; therefore, this patient was admitted to our surgical intensive care unit and received fluid resuscitation and empirical antibiotic treatment (meropenem against suspicious gram-negative bacteremia), although we could not determine the definitive infectious source of septic shock in this patient.

**Fig. 2:**
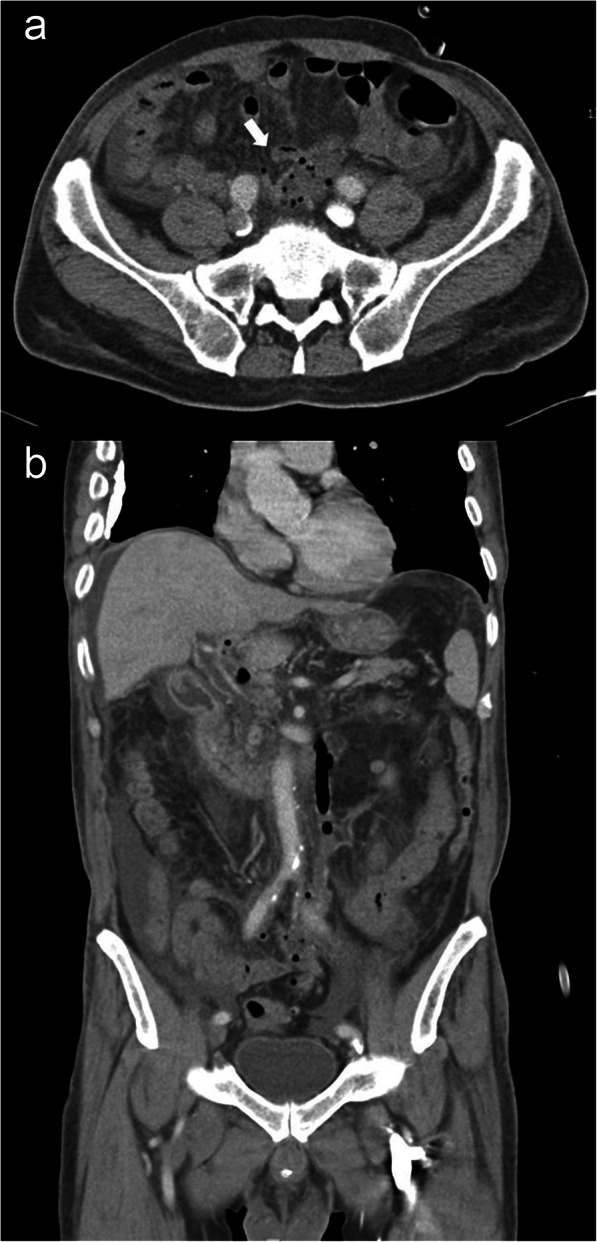
Follow-up CT showed a 4.1 × 4.0 cm RA (**a** arrow) and deteriorated thrombophlebitis of the portal venous system (**b**)

**Fig. 3:**
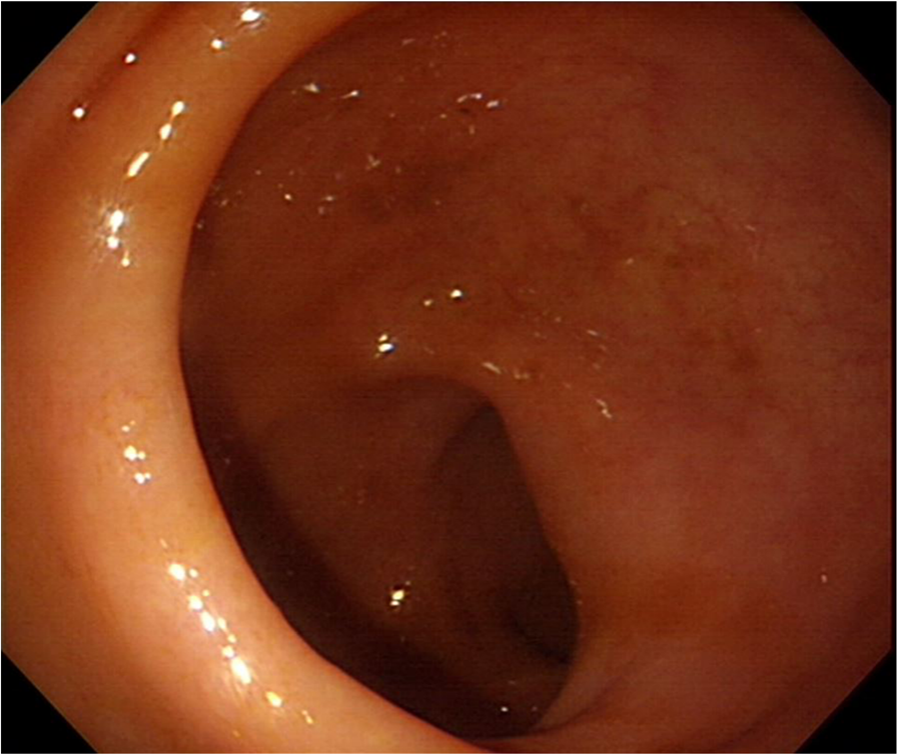
Preoperative sigmoidoscopy did not show any diverticular disease

**Fig. 4:**
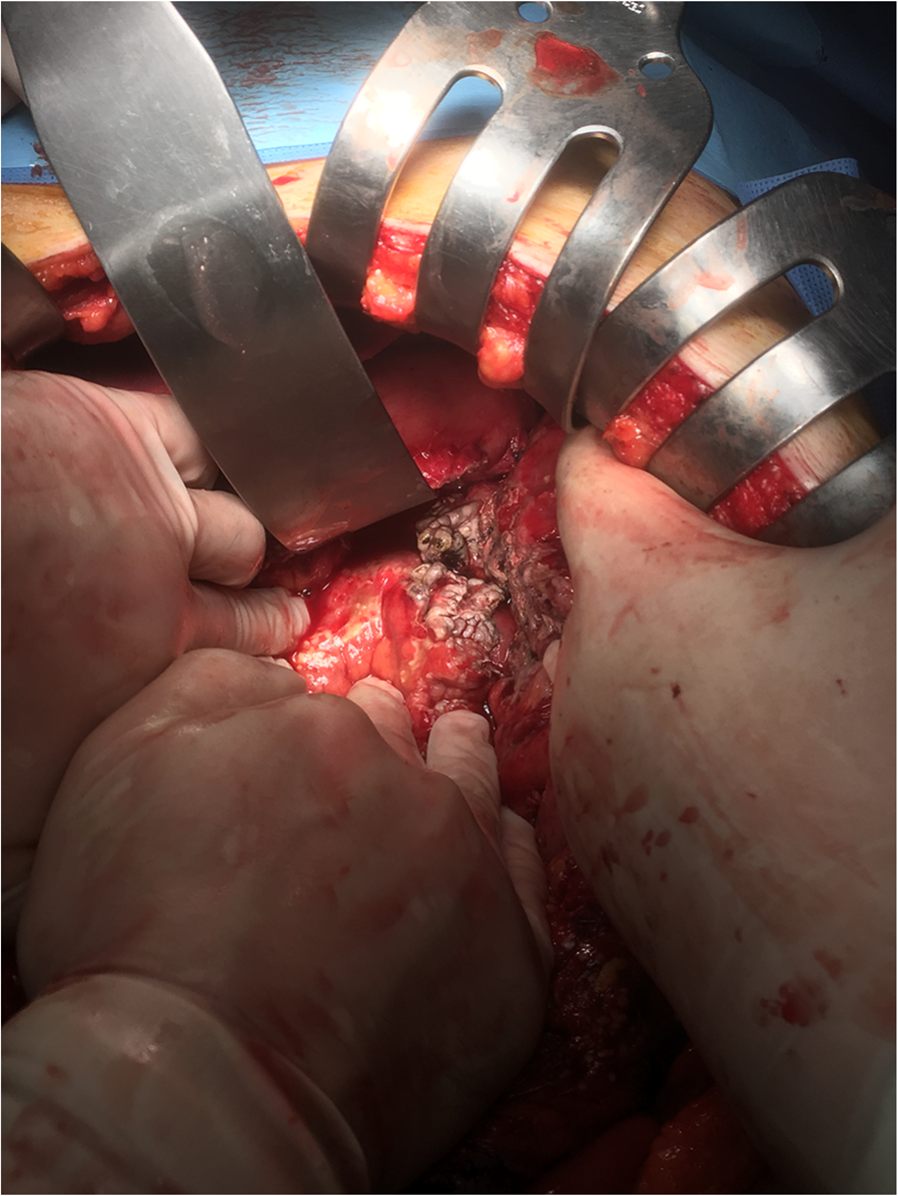
In the operative field, retroperitoneal dissection revealed a whitish abscess pocket around the iliac artery bifurcation

After admission to the intensive care unit and 5 days of hospitalization, the patient became hemodynamically stable, and the laboratory findings of jaundice and renal function were improved; however, he complained of persistent abdominal pain. As the renal function of the patient improved, we performed a consecutive abdominal CT scan with contrast enhancement to identify any possible cause of the abdominal pain in this patient and to recheck for any infectious origin of the sepsis. In contrast with the initial CT scan, the second CT scan showed a 5 cm abscess in the mesentery of the sigmoid colon and aggravated pylephlebitis of the portal venous system (Fig. 2a and b). Additionally, *Escherichia coli* (*E. coli*) bacteremia was confirmed via a blood culture test performed at the time of admission; therefore, the diagnosis of this patient was changed to sigmoid colon diverticular perforation with pylephlebitis based on both initial and consecutive CT scans. We performed sigmoidoscopy to identify the diverticula of the sigmoid colon; however, no diverticular disease was found (Fig. 3). As the results of the diagnostic work-up were conflicting and fatal outcomes were expected due to persistent pylephlebitis and abdominal pain, we performed an exploratory laparotomy on the 8th hospital day. The abdominal cavity was entered via a low midline incision. The peritoneal cavity was clean with no contamination, and we could not find any perforation or abnormalities in the biliary system or intestine, including the sigmoid colon. Instead, we observed an extraordinary retroperitoneal bulge, to which the sigmoid colon mesentery was attached, and retroperitoneal dissection revealed a 5 × 5 cm whitish abscess pocket around the iliac artery bifurcation (Fig. 4). This abscess cavity was completely separated from the sigmoid colon mesentery, and the capsule was severely adhered to the aorta and left iliac artery. The capsule was a solitary abscess with no fistula with adjacent organs. As the complete removal of the abscess cavity required massive dissection and might have caused unpredictable complications, we performed only incision, drainage, and curettage. A microbial culture test was also performed on the tissue and pus of the abscess cavity. Finally, an additional examination of the whole peritoneal cavity and organ systems was conducted; however, no infectious origin of the RA was found in the gastrointestinal or genitourinary system.

After the operation, microbial blood culture tests were performed, and intravenous vancomycin was empirically added to the antibacterial regimen to cover methicillin-resistant *Staphylococcus aureus* (MRSA), which is one of the major pathogens isolated from RAs. After a detailed interview with his daughter, we found that this patient had received frequent acupuncture by an unqualified therapist in the low lumbar region to treat chronic back pain, and the point of the acupuncture was exactly identical to that of the RA. The clinical and laboratory parameters of the patient gradually improved, and oral feeding was resumed on the 4th postoperative day. MRSA was identified in the microbial culture tests of pus and tissue; however, a blood culture test performed immediately after the surgery revealed no pathogen. In addition, there was no evidence of tuberculosis or malignancy on pathological examination for retrieved tissue. The patient responded well to the treatment and was discharged from the hospital at 20 days after the operation. We checked a follow-up CT scan taken in the outpatient department 40 days after the operation and identified no remaining pylephlebitis or abscess.

## Discussion and conclusions

RA can develop from various disease entities, including perinephric abscess, sigmoid colon perforation, postoperative abscess of the gastrointestinal tract, and puerperal diseases [1, 3–5]. The majority of the isolated pathogens are *E. coli*, *Clostridium* species, *Staphylococcus, Pseudomonas*, and anaerobes [1, 3, 6]. Among these, gram-negative bacilli are considered the most commonly isolated bacteria, and staphylococcal infections are mostly MRSA infections, which are usually associated with immunosuppression or bacteremia caused by remote infections [3, 5]. The overall incidence has not been reported to date; however, several studies have revealed that insidious and occult presentation without typical symptoms can cause difficulties in prompt diagnosis and accurate treatment [1, 3, 5]. Although the prognosis of RA has improved in recent decades, the mortality rate has varied from 1.5~15%, and surgical or percutaneous drainage with broad-spectrum antibiotics is considered to be the definitive treatment [1, 3].

To our knowledge, there has been no report on RA with pylephlebitis. Pylephlebitis, or infective suppurative thrombosis of the portal vein, is usually associated with pelvic infections, pancreatitis, inflammatory bowel disease, appendicitis, and sigmoid diverticulitis [7–9]. Pylephlebitis begins with the thrombophlebitis of small veins draining into an area of infection. The extension of the thrombophlebitis into larger veins leads to septic thrombophlebitis of the portal vein, which can extend further to involve the mesenteric veins. Surgical management is required when there is evidence of peritonitis, bowel infarction or perforation. In this case, the pylephlebitis on CT scan at admission caused diagnostic difficulty because the most common predisposing infections leading to pylephlebitis are diverticulitis and appendicitis [10]. Moreover, *E. coli* bacteremia supported the possibility of diverticular perforation as the cause of pylephlebitis in this patient. However, sigmoidoscopy showed normal intestinal mucosa, and this finding led us to perform a diagnostic laparotomy. The RA was eventually diagnosed after the operation, and the cause of the RA was thought to be frequent lumbar acupuncture because there were no infectious origins in the peritoneal and retroperitoneal space that could cause RA. In addition, the point of the acupuncture was exactly identical to that of the RA. Acupuncture is commonly and importantly used in traditional medicine in Korea and China and even in western countries as a tool for treating chronic pain, such as low back pain, osteomyelitis and migraine [2]. We carefully suggest that inappropriately performed acupuncture can cause unexplained soft tissue infections.

After retrospectively reviewing the patient’s medical records, we speculated the following: 1) the RA involved adjacent mesenteric veins and might cause pylephlebitis; 2) the prognosis might be fatal without infectious source control and changes in the antibiotic regimen (changing meropenem to vancomycin against MRSA); and 3) both *E. coli* and MRSA could be pathogens for septic shock in this patient because the bacteremia associated with pylephlebitis is frequently polymicrobial. Although it is still unclear why the results of the microbial culture tests showed discordances, the causative pathogens remain unidentified in approximately one-third of patients with sepsis [11]; therefore, it was possible that MRSA was not isolated in the initial and postoperative blood culture tests despite being a causative pathogen. Furthermore, *E. coli* might not be isolated on intraoperative abscess culture tests due to empirical meropenem treatment.

This case is the first report of RA caused by acupuncture and the first case of primary RA presenting with extensive thrombophlebitis up to the proximal portal system. Adequate source control is mandatory for RA, and early surgical intervention should be considered whenever the patient does not respond to broad-spectrum antibiotic treatment. In addition, RA can be suspected as the cause of pylephlebitis unless other pathologic conditions, including appendicitis, diverticulitis, and hepatobiliary sepsis, are diagnosed. Acupuncture practitioners should be aware of the potential for this procedure to have harmful adverse effects, as it may be a possible cause of otherwise unexplained soft tissue infections, especially in Asian countries.

## Data Availability

All patient data and clinical images obtained are contained in the medical files of Bucheon St. Mary’s Hospital, Korea. The datasets used during the present study are available from the corresponding author on reasonable request.

## Abbreviations

CT: Computed tomography
MRSA: Methicillin-resistant *Staphylococcus aureus*
RA: Retroperitoneal abscess

## References

[1] Crepps JT, Welch JP, Orlando R (1987). Management and outcome of retroperitoneal abscesses. Ann Surg.

[2] Yin C, Buchheit TE, Park JJ (2017). Acupuncture for chronic pain: an update and critical overview. Curr Opin Anaesthesiol.

[3] Coelho RF, Schneider-Monteiro ED, Mesquita JL, Mazzucchi E, Marmo Lucon A, Srougi M (2007). Renal and perinephric abscesses: analysis of 65 consecutive cases. World J Surg.

[4] Wataganara T, Sutantawibul A, Anuwutnavin S, Leelaporn A, Rongrungruang Y (2014). Puerperal retroperitoneal abscess caused by *Clostridium difficile*: case report and review of the literature. Surg Infect (Larchmt).

[5] Yamamichi F, Shigemura K, Kitagawa K, Arakawa S, Tokimatsu I, Fujisawa M (2017). Should we change the initial treatment of renal or retroperitoneal abscess in high risk patients?. Urol Int.

[6] Brook I, Frazier EH (1998). Aerobic and anaerobic microbiology of retroperitoneal abscesses. Clin Infect Dis.

[7] Bazan F, Busto M (2015). IMAGES IN CLINICAL MEDICINE. Pylephlebitis as a complication of diverticulitis. N Engl J Med.

[8] Ram P, Lapumnuaypol K, Punjabi C (2017). Diverticular pylephlebitis and polymicrobial septicemia. Case Rep Infect Dis.

[9] Yazgan C, Akkas M, Ozmen MM (2015). Inferior mesenteric vein pylephlebitis due to sigmoid diverticulitis. BMJ Case Rep.

[10] Plemmons RM, Dooley DP, Longfield RN (1995). Septic thrombophlebitis of the portal vein (Pylephlebitis): diagnosis and management in the modern era. Clin Infect Dis.

[11] Rhodes A, Evans LE, Alhazzani W, Levy MM, Antonelli M, Ferrer R (2017). Surviving Sepsis campaign: international guidelines for management of sepsis and septic shock: 2016. Intensive Care Med.

[12] Lee H, Cho J, Sung K (2017). Fatal retroperitoneal abscess caused by lumbar acupuncture. Abstract book of 69^th^ annual congress of Korean surgical society.

